# Smart Composite Materials with Self-Healing Properties: A Review on Design and Applications

**DOI:** 10.3390/polym16152115

**Published:** 2024-07-25

**Authors:** Artemis Kontiza, Ioannis A. Kartsonakis

**Affiliations:** Laboratory of Physical Chemistry, School of Chemistry, Aristotle University of Thessaloniki, 54124 Thessaloniki, Greece

**Keywords:** microcapsules, self-healing, thermoplastic polymers

## Abstract

Research on self-healing materials spans multiple academic disciplines and employs a variety of methodologies. Nature has been a major source of inspiration for developing self-healing materials and will likely continue to inspire innovative ideas in this field. This review article covers the principles of self-healing mechanisms, focusing on both autonomous and non-autonomous procedures. It explores both intrinsic and extrinsic self-healing abilities by considering their components, structures, and design. Additionally, a detailed analysis of the application of these materials across various sectors is provided, including aerospace, automotive, marine, energy, medical and healthcare, military, and construction. Finally, the review paper highlights the advancements in encapsulation technologies for microcapsules, their thermal stability, their mechanical properties, and the compatibility of healing agents with the matrix, which play a crucial role in the effectiveness of self-healing processes.

## 1. Introduction

Smart composite materials, also referred to as adaptive or intelligent composites, are advanced materials designed to respond to environmental stimuli in a useful and predictable way [1]. Intelligent systems that encompass machine learning and artificial intelligence technologies have a wide range of applications across different fields, including coatings, manufacturing, education, energy, agriculture, finance, and cybersecurity. The development of materials capable of performing various functions and responding to external stimuli is expected to be a significant research focus in the foreseeable future. These innovative materials will be essential in fields such as additive manufacturing, as they can be designed and structured to execute specific tasks and autonomously adapt to changing external conditions. Such materials cater to the demands for multifunctionality and adaptability, simplifying system complexity and facilitating easier implementation of solutions. Traditional composite materials typically consist of a polymer, ceramic, or metal matrix that binds the material or includes reinforcements like particles or fibers that enhance stiffness and strength. Smart materials encompass piezoelectric materials, magnetostrictive materials, shape memory alloys, shape memory polymers, light-sensitive materials, hydrogels, electrochromic and thermochromic materials, electroactive polymers, magnetorheological and electrorheological materials, carbon nanotubes, graphene, and self-healing polymers [2,3].

**Figure 1:**
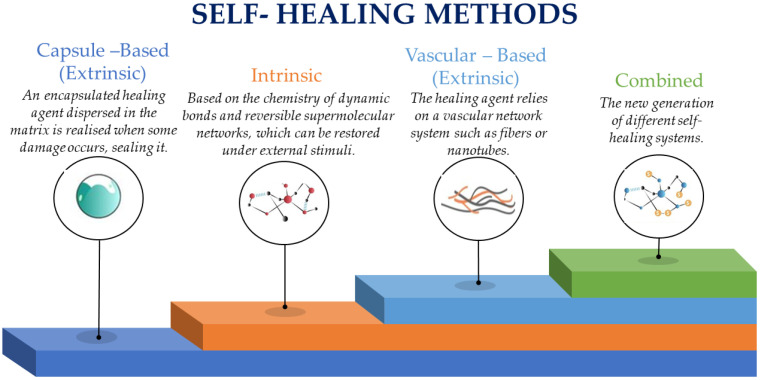
The self-healing methods.

Self-healing materials (materials with self-healing abilities) are a category of smart composite materials that can automatically repair their damage and restore their original functionality and properties after being damaged [4]. The self-healing material types are mostly polymeric, ceramic, metallic, and the combination of them (composite materials). Table 1 summarizes the main characteristics and application areas of different types of self-healing materials. Polymeric materials with self-healing abilities can be manufactured via microencapsulation and intrinsic healing [5]. On microencapsulation, capsules including healing agents are loaded in the material. When damage formation occurs, the healing agent releases from the capsules and ruptures, resulting in the repairing of the crack. Intrinsic healing is based on polymers that have reversible bonds that can be reconstructed after being broken due to an external triggering such as light, heat, or pressure. Metallic materials’ self-healing is based on self-healing alloys that can undergo phase transformations, allowing them to clog cracks and reconstruct their structure, and on healing via heat treatment where metals are designed to heal via procedures such as diffusion and recrystallization after heat treatment [6]. Ceramic materials’ self-healing effect is based on crack healing ceramics where new bonds at the crack surfaces are formed when exposed to high temperatures, resulting in a healing outcome [7]. The composite materials’ self-healing effect is based on vascular systems and on shape memory alloys [4,8]. In the vascular systems, the composites contain networks of hollow tubes loaded with healing agents. When damage occurs, the healing agent is released to the damaged area. In shape memory alloys, the composites utilize shape memory effects to clog cracks and reobtain functionality (Figure 1).

Recently, a noticeable gap has developed between intrinsic self-healing materials and self-healing composites based on capsules. Research on capsule-based systems primarily focuses on the rupture process, mixing procedures, healing agents, and micro-structure manufacturing techniques. The initial work centers on three main self-healing methodologies, addressing challenges and key issues associated with each approach. This review provides an overview of the literature on healing agents used in recent years. The article explores recently developed distinct self-healing methods, many of which are inspired by biological systems. The focus here is on self-healing thermoplastic polymers that can also be used as composite filaments [9]. Taking into account that a novel technique of producing three-dimensional (3D) physical objects from three-dimensional CAD (computer-aided design) data is 3D printing, composite filaments with self-healing abilities that can be used for 3D printing represent a development in additive manufacturing, combining the advantages of self-healing capabilities with composite materials.

Previously published review papers have extensively covered various aspects of self-healing materials, such as the mechanisms of self-healing, the types of materials used, and specific applications in different fields. However, this review paper differentiates itself by focusing on the recent advances in the design and application of self-healing agents, particularly microcapsule-based systems and self-healing thermoplastic polymers.

The novelty of this review lies in its comprehensive examination of the integration of self-healing mechanisms in smart composite materials, with a particular emphasis on autonomous self-healing properties. This paper explores both intrinsic and extrinsic self-healing abilities by considering their components, structures, and design. Additionally, this paper provides a detailed analysis of the application of these materials across various sectors, including aerospace, automotive, marine, energy, medical and healthcare, military, and construction. This review paper also highlights the advancements in encapsulation technologies for microcapsules, their thermal stability, their mechanical properties, and the compatibility of healing agents with the matrix, which play a crucial role in the effectiveness of self-healing processes.

## 2. Self-Healing Mechanisms

**Figure 2:**
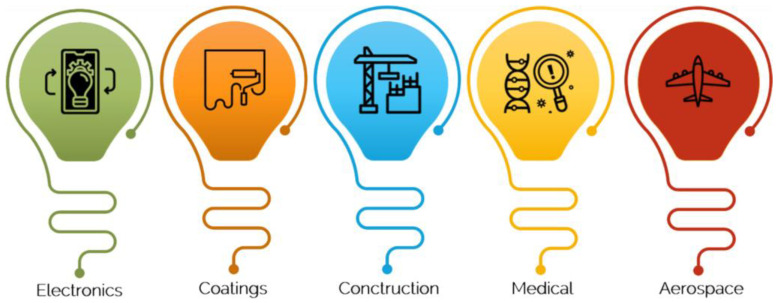
Self-healing technology integration into diverse applications.

Self-healing is a method characterized by its ability to autonomously identify malfunctioning devices or systems [10,11]. This mechanism is designed to repair microcracks in systems, thereby enhancing their mechanical or functional performance. It signifies a new era of technology, significantly enhancing the crucial performance aspects of various products. Self-healing technology has been incorporated into various applications, including structural, electronic, medical, and aerospace products, enhancing their overall performance (Figure 2). This technology is especially beneficial in scenarios where repairs or inspections are difficult, dangerous, and expensive [12].

### 2.1. Autonomous and Non-Autonomous Mechanisms

Autonomous mechanisms can repair their structural integrity or functional properties without any external intervention, as the damage itself triggers the repair processes. In contrast, coatings that are not inherently self-healing rely on external stimuli, typically light or heat, to initiate the healing process. Unlike autonomous self-healing, this process can be repeated. To enhance the response to stimuli, complementary corrosion-sensitive components that detect pH changes during the early stages of corrosion can be added to these coatings [13]. The healing of these coatings is facilitated by the reformation of the polymer network’s intrinsic chemical bonds and/or physical structure within the coating matrix, which improves chemical stability and provides a self-healing option for protective coatings on magnesium alloys. As discussed below, the reaction mechanisms involve shape memory effects and dynamic bonding [14].

Reversible physical or chemical polymer networks that can mitigate coating defects are essential to the non-autonomous self-healing process. However, until artificial external stimulation is applied, these defects remain exposed to corrosive conditions. Non-autonomous (or stimuli-assisted) healing systems can repair coatings using external stimuli unrelated to corrosion events. Common stimuli for this type of coating include heat, light, mechanical forces, chemical reactions, and pH changes, as they can be easily applied in service environments. The properties of the polymer coatings primarily determine the effectiveness of non-autonomous smart self-healing anti-corrosion coatings. When stimulated by heat or light, the polymer coating can directly repair itself, significantly reducing the need for external repair chemicals. Theoretically, the coating can be repaired an infinite number of times. Non-autonomous healing coatings have a greater potential to quickly mend cracks, especially those covering large areas, compared to autonomous self-healing coatings. However, some protective coatings are expensive, have limited application ranges, may pose environmental risks, and exhibit poor mechanical properties and low fracture resistance.

### 2.2. Intrinsic and Extrinsic Mechanisms

Autonomous mechanisms can be reported from two perspectives: intrinsic and extrinsic self-healing mechanisms [15]. This review explores the classifications of self-healing materials commonly used in various applications, specifically focusing on intrinsic and extrinsic self-healing mechanisms. These types are further divided into subdivisions based on criteria such as mode of contact, healing agents, and catalysts, which are discussed in subsequent sections. The final section of the article examines recent developments and applications of self-healing materials across different fields.

Broadly, self-repair processes in self-healing materials fall into two main categories: extrinsic healing and intrinsic healing. Extrinsic healing features include microcapsules, microvascular networks, and nanoparticles. Intrinsic healing, on the other hand, is divided into dynamic covalent bonding, such as Diels–Alder reactions and disulfide formation, and dynamic non-covalent bonding, which involves hydrogen bonds, ionic interactions, and coordination [16].

One category of extrinsic healing behavior involves bacteria-assisted crystal precipitation, which has been successfully applied to restore cementitious materials. This innovative technology is also used to heal structured ceramics [17,18].

Polyurethanes and polyurea have the potential to create inherently recoverable polymers due to their hydrogen bonds [19,20,21]. These highly dynamic hydrogen bonds can be leveraged to design intrinsic self-healing materials. Leibler and his research team demonstrated the self-healing effects arising from the dynamic nature of hydrogen bonds. Furthermore, the shape-memory properties of polyurethanes can enhance the self-healing characteristics of polymers, a phenomenon known as shape-memory-assisted self-healing [22,23,24].

Metal corrosion can trigger the release of inhibitive species in coatings, which serve to halt the progression of corrosion, a process known as “active protection” [25,26]. This involves passivating the defective area with active species, allowing for the full or partial recovery of the flawed region by safeguarding the exposed metal from further corrosion. Two distinct autonomous self-healing mechanisms may be involved: (1) the formation of a protective layer primarily through the oxidation of the healing agent or related reactions to fill defects, and (2) the generation of chelates or precipitates associated with corrosion inhibitors to block the corrosion path [27,28]. Mechanical damage can release corrosion inhibitors, often used in coatings with autonomous self-healing mechanisms that fill cracks.

Cracking is a significant issue in polymer composites due to the contact between a thin, brittle layer and a deformable layer. The study of microcrack formation and propagation is crucial for understanding a material’s ability to withstand stress without failing during application, closely linked to the material’s resilience. This becomes more complex in polymer composite materials, where the distinct properties of the reinforcement and the matrix play a role. Polymer composites undergo mechanical loadings and environmental factors during fabrication, storage, and service, leading to the development of microcracks under various loading conditions, including tension, compression, and shear [29].

Exposure to fluctuating environmental conditions, such as temperature, moisture, chemicals, and radiation, also contributes to microcrack formation and propagation. Polymer composites subjected to both mechanical loading and environmental exposure are generally more prone to microcrack formation and propagation. The immediate occurrence of microcracks in polymer composites results in the deterioration of thermomechanical properties and serves as an initiator for other types of damage, such as delamination, fiber–matrix interfacial debonding, and fiber fracture. This provides pathways for moisture, oxygen, and other corrosive liquids, leading to overall material degradation and significantly impacting the long-term durability of polymer composite materials [30,31].

## 3. Microcapsules’ Synthesis Process

A variety of repair agents can be encapsulated to mend polymer damage. To ensure effectiveness during the repair process, the capsules’ shells must possess high thermal stability and appropriate mechanical properties [32,33]. Additionally, the catalyst must have high thermal stability and good solubility in the repair agent. Key factors that impact the construction and enhancement of the self-healing performance of polymeric materials using microcapsules are also considered. This paper explores recent advancements in intrinsic repair systems, particularly their application in polymer composites. The review addresses the need for composite repair, the self-healing concept, various repair methods, evaluations of repair performance through different mechanical tests, and statistical insights and trends related to self-healing.

Capsule-based smart composite materials were an emerging field of research and development [34]. These materials hold great promise due to their ability to incorporate microcapsules containing functional agents within a composite material. These functional agents could be used to provide various properties or functionalities to the composite material, such as self-healing, sensing, or controlled release of substances. Extrinsic and intrinsic mechanisms are two primary divisions of self-healing polymers, which are categorized according to the type of healing. In both cases, a mobile phase that can fill the crack during the healing process is essential. For intrinsic self-healing, this can be achieved through the presence of reversible covalent bonds. For extrinsic self-healing, the mobile phase can be provided by the flow of integrated catalysts or thermoplastic additives. Some sources also mention the inclusion of a secondary phase, which can enhance the material’s self-healing capabilities and influence its engineering performance.

### 3.1. Micro and Nanocapsules

Nano/microcapsules are generated using various methods in a variety of structural shapes (single-core, multi-core, multi-core, irregular) and possess a multitude of useful qualities that are useful for a variety of applications. The morphologies include (a) the shell reducing the core’s reactivity, (b) the thermal stability of the dispersibility and stability of the particles is improved overall, and the particles being easily modifiable. The micro-nano/core-shell capsules have excellent interest because they have remarkable qualities resulting from the combination of core and shell material and their geometrical structure. As a result, they have discovered broad application in domains such as catalysts, biology, and electrical and semiconducting materials. Additionally, core-shell nanoparticles stand out as the most promising and suitable morphology for medical applications such as drug delivery, gene delivery, and sensors, thanks to their superior dispersibility, shape, optical properties, and bio-functionalization [35].

### 3.2. Encapsulation Technologies for Micro/Nanocapsule Fabrication

**Figure 3:**
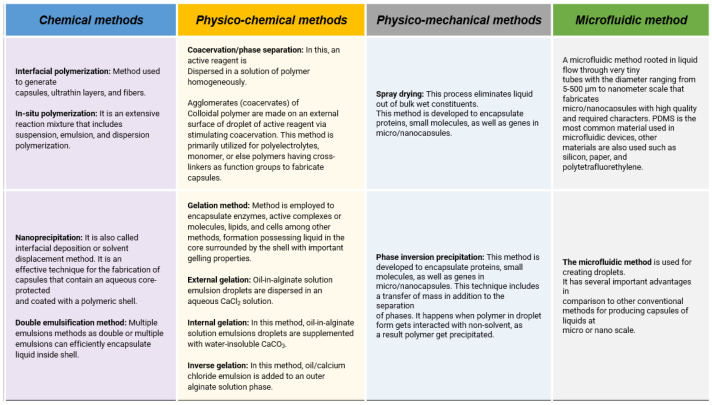
Tabulated methods for microcapsules synthesis together with active reagents encapsulation.

Many capsules exhibit diverse morphologies depending on the fabrication material, with the synthetic method also significantly influencing the final product. Chemical methods involve the creation of micro/nano spheres through various polymerization reactions, using monomers or prepolymers as the starting materials. When shaping capsules involves multiple physical transformations without polymerization processes, it is referred to as the physico-mechanical method, while those involving chemical reactions are known as the physico-chemical method. Traditional microencapsulation techniques have some limitations, including specific control over morphology, size distribution, composition, and porosity of the developed particles, a process referred to as the microfluidic method. (Figure 3) [36,37,38].

Double-walled microcapsules were synthesized using the general method described by Caruso et al. [39], which involves the single-batch synthesis of polyurethane/poly (urea-formaldehyde) (PU-UF) microcapsules. The process described by Shinde et al. [40] is double-shell-walled microcapsule synthesis via an in situ interfacial emulsion polymerization process for preparation of EPA-filled microcapsules for FFF printing. White et al. conducted the initial research on dicyclopentadiene (DCPD) as the healing agent to be encapsulated [41]. A Grubbs catalyst was used to successfully demonstrate self-healing in bulk materials and coatings, but it lacks practical application due to the catalyst’s poor chemical and thermal durability, poor matrix dispersion, and expensive cost. Examples of alternative healing agents that have been studied include epoxy resins, oil-based agents, and siloxanes. Epoxy resins, in particular, are considered key core materials due to their ability to react with a wide range of curing agents or hardeners, such as amines and anhydrides, at various temperatures. They also adhere strongly to numerous substrates and maintain thermal stability, with a high thermal decomposition temperature even at 200 °C. However, commercial epoxy resins often have high viscosity, necessitating the use of reactive diluents to reduce it. When used in bulk materials, epoxy resins have demonstrated self-healing effectiveness of over 90%. Multiple healing after the initial repair is only possible if an overheating agent is present in the matrix. To enable multiple healing in composite materials, a new reservoir capable of transporting larger quantities of liquid healing agents has been developed.

The team of Jung et al. [4], in order to evaluate self-healing systems, placed the self-healing agent into a polyoxymethylene urea storage container and then the containers were incorporated within a polyester matrix. The best healing results were achieved with a styrene-based system containing 1.3 wt% dimethylaniline (DMA), 1.3 wt% cobalt naphthenate, and 0.01 wt% paratertbutylcatechol (TBC). However, it was noted that this system had limited practical use due to the short shelf life of the healing chemicals. Jung’s system employed optical techniques, including optical microscopy, SEM, and high-speed video imaging, to identify the rupture process, where microcapsules released their contents into an emerging crack. Strong interfacial adhesion between the matrix and microspheres was crucial; although this initiated self-healing, it reduced the composite’s overall durability. Compared to plain polyester resin, self-healing samples showed increased fracture resistance at the cost of material rigidity. An alternative microencapsulation approach was patented by Skipor et al. [42] introducing the concept of attaching catalyst molecules to the exterior of microcapsules filled with the healing agent. Positioning the catalysts near the release site was claimed to potentially enhance overall healing efficiency. This patent proposed an improved approach by crosslinking the healing agent directly with the damaged surfaces, eliminating the need for a catalyst.

In the work of Yang et al. [43], a two-component healing system was used where UF capsules with a range of diameter 30–70 µm loaded with epoxy were distributed in a matrix containing a latent hardener. The latent hardener, a complex of CuBr2 with 2-methylimidazole, was well dispersed in the epoxy composite matrix. When mechanical stress induced microcracks, the UF spheres would break, releasing the epoxy to cure upon contact with the embedded hardener. They found that the self-healing epoxy with 10 wt% spheres and 2 wt% hardener achieved 111% of its original fracture toughness, while a composite with woven glass fibers achieved a healing efficiency of 68%. Polyurethane (PU) is often used as a healing agent, showing success in both durability and the recovery of mechanical properties. Maes, Van Tittelboom, and De Belie [44] studied the healing capabilities of PU in macrocapsules for resisting chloride environments. They found that 67% of specimens with PU nearly fully regained resistance to chloride penetration at a crack width of 0.10 mm, whereas only 33% showed no chloride ion penetration at crack widths increased to 0.33 mm.

Anglani et al. [45] reported in their work a healing rate of 35.9–46.5% when using PU as a healing agent, compared to only 0.1% for control specimens. Using larger diameter microcapsules (7.5 mm) with an epoxy coating led to a 50% regain in flexural load capacity upon first reloading and 82% upon the second reloading, attributed to the hardening of the PU. Haiyan Li et al. [46] developed polysulfone microcapsules containing tung oil using a solvent evaporation method. These microcapsules, with a mean diameter of 130 µm and wall thickness of 9 µm, showed high thermal stability with a thermal degradation onset temperature of 350 °C. Incorporating these microcapsules (10 wt%) into an epoxy matrix resulted in a multi-functional coating with self-healing and self-lubricating properties. The microcapsules exhibited excellent anticorrosion performance in scratched coatings, attributed to the formation of a cross-linked polymer film from released tung oil. The self-lubricating coating also showed a significant decrease in frictional coefficient and wear rate compared to neat epoxy.

In the research of Montemor et al. [47], the researchers used modified epoxy-based coatings with microcapsules loaded with isophorone diisocyanate and pH-sensitive Ce tri(bis(2-ethylhexyl)phosphate) (Ce(DEHP)_3_) particles as inhibitors. This healing process involved multilevel protection and synergistic effects that significantly reinforced the corrosion protection conferred by the modified epoxy coating. Ye et al. [48] synthesized graphene sheets containing a porous polyhedral oligomeric silsesquioxane (POSS) framework as nanocontainers for self-healing organic coatings. The obtained porous graphene sheets were loaded with Benzotriazole (BTA) and embedded into the epoxy coating, forming a composite coating. The BTA released from the nanocontainer formed an adsorption layer on the metal surface, and the physical barrier of graphene suppressed the permeation of the corrosion medium. In the study of Chen et al. [49], the researchers synthesized TiO_2_ nanotubes using a hydrothermal method to act as nanocontainers for molybdate corrosion inhibitors. The molybdate-loaded TiO_2_ nanotubes were pH-sensitive, and the fabricated PPy/TiO_2_@Mo coating exhibited superior corrosion resistance and electroactivity. The improved anti-corrosion performance was attributed to the anodic protection, physical barrier, and efficient release of corrosion inhibitors from the TiO_2_ nanotubes. Shchukin et al. [50] investigated the corrosion-protection ability of a developed coating based on a combination of passive and active parts loaded with nano- and microcontainers. The active part included polyurethane microcontainers loaded with alkoxysilanes with a long hydrophobic tail, revealing improved corrosion protection of the formed film.

### 3.3. Self-Healing Thermoplastics

A type of polymer known as thermoplastics solidify upon cooling after reaching a certain temperature, at which they become malleable or moldable. Due to their intrinsic flexibility, ease of processing, and recyclability, these materials are especially appealing as matrices for composites capable of healing themselves. Reversible bond forms and phase transitions that occur in reaction to external stimuli like heat, light, or pressure are responsible for thermoplastics’ capacity for self-healing. Advances in self-healing thermoplastics have demonstrated great promise in extending the life cycle and durability of composite materials used in various industries, including aerospace and automotive [51].

Self-healing composites, for example, are valuable for the aerospace industry in crucial components where repair and maintenance are challenging and costly. Similar to this, self-healing materials in the automobile sector can extend the life and safety of vehicle components, lowering the frequency of replacements and repairs. Researchers have created several novel strategies to improve thermoplastics’ capacity for self-healing, even though there are still several obstacles in this field, despite promising improvements. These include ensuring that manufacturing processes are scalable, integrating the requirements of the environment with the usage of certain chemicals and nanomaterials, and improving the trade-off between healing efficiency and mechanical characteristics. Subsequent investigations are anticipated to concentrate on creating more effective healing processes, improving the eco-friendliness of self-healing materials and broadening their uses in many sectors of the economy. Self-healing thermoplastics have the ability to transform material design and greatly increase the life cycle of composite materials if they can overcome these obstacles [52].

## 4. Design of Self-Healing Materials

Inducing or designing self-healing mechanisms in rigid materials below their glass transition temperature (T_g_), such as composite matrices, presents a significant challenge. This is particularly important for glassy amorphous polymers, semicrystalline materials, and thermosetting composite matrices, which are often extensively cross-linked. C.M. Dry’s research team [53] developed several patents on advanced materials with self-healing properties, particularly fiber-filled composites, which have applications in concrete and polymer matrices. They created a system of hollow fibers containing a reactive fluid that, when subjected to mechanical shock, was released to seal fissures. Dry et al. [54] conducted several self-repair tests on polymer matrices with various fibers. One notable experiment involved polymer samples with an embedded continuous metal fiber and two adjacent self-repair fibers—one containing an epoxy monomer and the other a diamine cross-linking agent. During a fiber-pullout test, they initially unbonded the metal fiber mechanically without affecting the self-repair fibers. At this point, the metal fiber had minimal adhesion to the matrix and was easily removed. Sub-critical loads applied to the composite allowed the fluids in the fibers to mix, diffuse to the polymer–metal interface, and repair the damage, significantly increasing the fiber pullout stress. This self-repair approach is versatile, allowing for variations in fiber design, number of fibers, healing fluids, fiber construction, and fiber coatings.

Matrix solvents can also be used to promote solvent bonding in micro-voids. Thermally induced healing can occur through exothermic reactions of the self-repair fluid, either with itself or with composite components [55]. When considering self-healing, it is essential to account for the topography or roughness of the surface and how it changes with time, temperature, and pressure after contact with the healing fluid. The rate of crack healing in fractured polymers is influenced by changes in fibrillar shape and other variables. Molecules diffusing back into the bulk can alter chain-end distributions near the surface. If chain ends are needed for fluid interaction, they can be encouraged to migrate to the surface by using moieties with lower surface tension. Molecular weight distribution may also change spatially, with low molecular weight species migrating to the surface more frequently. Nanoparticles in the bulk might preferentially move into nanovoids. In time-release solvents or adhesives, polymer–solvent interactions affect surface rearrangement. Surface chemical reactions, such as oxidation and cross-linking, can complicate diffusion dynamics. Solvents used in passive healing experiments might also cause additional damage by swelling crazes and microvoids, allowing them to propagate further [56]. The critical entanglement molecular weight Mc will also change with surface polymer concentration φ in a good solvent as:

M_c_(φ) = Μ_c_(1)φ^−5/4^ (1)

where M_c_(1) is the unperturbed M_c_ value with φ = 1.

This indicates that interaction with a compatible healing fluid increases the entanglement molecular weight.

In polymer–solid interfaces common in composites, surface restructuring dominates adhesion mechanisms between the polymer and the solid. Using a mole fraction of sticker groups f(X) to bond the polymer to the surface, an optimal sticker group concentration f*(X) maximizes adhesion while minimizing cohesive failure in the boundary layer adjacent to the solid [57]. When f < f*, adhesive failure dominates, and the solid separates cleanly from the polymer, with fracture energy G_1c_ proportional to f. When f > f*, cohesive failure occurs in the polymer layer adjacent to the surface, and G_1c_ is inversely proportional to f. The optimal f* value is derived from the entanglement percolation theory as:

When f < f*, adhesive failure dominates, the fracture energy G_1c_~f and the solid separates cleanly from the polymer. When f > f*, cohesive failure occurs in a polymer layer adjacent to the surface and G_1c_~1/f. The f* value is determined from the entanglement percolation theory to be:

(2) $$f^{*}=4j\left(\frac{M_{j}}{M_{c}}\right)$$

where M_j_ is the molecular weight per bond of a random walk chain.

The surface technique involves treating crack surfaces brought together to heal, either independently or with the assistance of a healing fluid. This stage considers the time-dependent contact of the various surfaces to construct the interface. In composites, where damage may affect the polymer matrix, fibers, and the matrix–fiber interface, the surface approach is crucial. Healing will be minimal or nonexistent if the healing fluid only adheres to one surface. The pressure of swelling can also help press the surfaces together during the healing process.

Self-healing composite filaments for 3D printing allow the fabrication of complex and intricate structures with relative ease. The production of these filaments for 3D printing includes several considerations and key steps to ensure the material’s compatibility and effectiveness with additive manufacturing processes. The selection of self-healing material, the composite filament formulation, the manufacturing process, the printing considerations, the testing and validation, the scaling production, the application and potential uses, and finally the safety and environmental considerations are the important steps for the development of self-healing composite filaments that can be used in 3D-printed applications [58]. Fused filament fabrication (FFF), a form of material extrusion, is the most widely used and accessible technology due to its low cost, quick cycle times, and ease of use. The FFF extrusion manufactures objects from 3D CAD models via thin layers of extruded thermoplastic filaments that have been semi-melted [10,59,60]. Ameri et al. examined the self-healing capability of 3D-printed polymeric composites reinforced by shape memory alloys and epoxy as healing agents [61]. In the work of Balla et al., composite filaments from natural fibers like bamboo, flax, and hemp were fabricated and used in 3D-printed operations [62]. The study of Ning et al. was based on the combination of acrylonitrile butadiene styrene with carbon fiber during the FFF process [63]. The investigation of Tian et al. includes the production of carbon fiber and PLA filament as reinforcing phase and matrix, respectively, that were utilized in 3D printing applications [64].

## 5. Damage and Healing Theories

**Figure 4:**
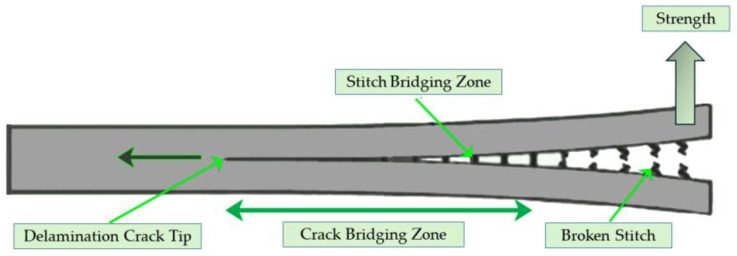
The microscopic entanglement structure.

By examining the damage mechanisms, materials can inform us about their own healing processes. Figure 4 illustrates a practical method for calculating the fracture energy G_1c_ of an A-B polymer healing interface [65]. In the double-cantilever beam (DCB) system, a crack initially forms at the crack tip and then propagates through the interface region. The J-integral approach can be used to calculate the fracture energy for cohesive failure. Here, G_1c_ is the integral of the traction stresses with crack opening displacements δ, in the cohesive zone after yielding at a local yield or craze stress σ_γ_. At a maximum stress value, the cohesive zone at the fracture tip disintegrates through a percolation process (at a maximum stress value, σ_m_ > σ_γ_). The yield stress dominates the fracture process for non-crazing matrices such as thermosets and is determined by the twinkling fractal theory (TFT). Both σ_m_ and δ are rate-dependent and, in the simplest case, are determined by:

G_1c_ = σ_m_ δ_m_ (3)

where δ_m_ is the critical crack opening displacement. Both σ_m_ and δ_m_ depend on the damage zone structure and the microscopic deformation mechanisms controlling the percolation fracture process via disentanglement and bond rupture.

The fracture and healing on any 2D or 3D lattice are subject to random bond fracture, starting with an initial tensile modulus E. Microvoids are created by random bond scission, which then expand into larger voids facilitating the propagation of a macroscopic break across the network at the percolation threshold. The stored elastic strain energy density ρ, (energy per unit volume), in the lattice due to an applied uniaxial stress σ is determined by:

(4) $$\rho =\frac{\sigma^{2}}{2^{Ε}}$$

In several recent papers, Boiko et al. [66] investigated the bonding of virgin rather than fragmented surfaces to determine healing at the PET and PS interfaces. It was shown that even after 15 h of treatment at 18 °C above their T_g_, virgin PET/PET and PET/PS joints exhibited minimal adhesion. The team of Yang F. et al. [67] examined the interfacial healing of carbon-reinforced polyether–ketone–ketone (PEKK) and polyether–ether–ketone (PEEK) under non-isothermal conditions. They compared the strength of thermally bonded plates with their ultimate shear strength after various processing times. All tested systems achieved 100% efficiency, and a model for the non-isothermal healing of thermoplastic surfaces was proposed, although this model seems more suited to polymer processing than to repair.

Takeda et al. [68] demonstrated that various technical thermoplastics made through condensation processes, such as polybutylene (PB), polycarbonate (PC), polyether–ketone (PEK), polybutylene terephthalate (PBT), and PEEK, exhibit a rapid response to chain scission reversal. They observed that the self-healing reaction in these polymers occurred in the solid state, identifying a series of events before and during the healing process: (i) chain cleavage due to degradation; (ii) diffusion of oxygen into the polymer materials; (iii) recombination of cleaved chain ends via a catalytic redox reaction under an oxygen atmosphere and in the presence of a copper/amine catalyst; and (iv) water discharge resulting from the self-healing reaction.

Additionally, it was observed that as the reaction time increases, the speed of the healing response decreases due to reduced mobility of the polymer chain. This occurs as the reaction progresses with increasing molecular weight and a gradual depletion of available hydroxyl (OH) end groups consumed during recombination. Various techniques were used to elucidate the self-healing mechanism, including peel tests, controlled projectile tests, scanning electron microscopy (SEM), differential scanning calorimetry (DSC), thermogravimetric analysis (TGA), and a pressurized burst test to measure the healing response.

A common approach among researchers to study self-healing is by observing the healing of crack width [69]. Numerous studies in the technical literature indicate that crack size is a crucial factor in autogenous healing. Recent research reported that larger cracks, specifically those 0.40 mm wide, could not be completely healed; specimens with 0.40 mm cracks showed incomplete healing [70]. Therefore, as crack width increases, the effectiveness of autogenous self-healing diminishes, making complete healing unlikely. Even for narrower cracks, the healing process may only be partially effective [71]. Specifically, crack widths between 0.138 mm and 0.150 mm have been reported to only partially heal.

The challenge in designing self-healing materials lies in creating new composite materials with autonomous or externally stimulated damage-healing capabilities to extend their performance lifetime. This definition also encompasses thermal or electrochemical degradation. Monitoring these changes is essential to evaluate the performance of the new composite material. These modifications can help describe the healing process qualitatively or statistically. Ideally, the new material’s properties will be equal to or better than those of the original. To assess the performance of the improved self-healing material, researchers or engineers should compare it to the virgin, unmodified material [72].

Most self-healing systems reported in the literature consist of a polymeric matrix and self-healing agents. The most frequently used characterization techniques for these materials include Dynamic Mechanical Analysis (DMA), Differential Scanning Calorimetry (DSC), and Thermogravimetric Analysis (TGA). DMA is widely used to determine the glass transition temperature of the constituent materials and to assess viscoelastic properties in terms of storage and loss moduli. TGA is used to determine characteristics of materials that exhibit mass gain or loss. Other techniques employed in the characterization of self-healing materials include Fourier Transform Infrared Spectroscopy (FTIR), Nuclear Magnetic Resonance (NMR), and RAMAN spectroscopy. These techniques are extensively used for monitoring the self-healing process and will be discussed further in later sections [73,74,75]. The self-healing concept has also been successfully implemented for materials with electrical functionality, enabling them to recover conduction paths at different scales. Most studies on conductivity recovery focus on healing these conductive paths. In their self-healing studies, Blaiszik et al. [76] used a Wheatstone bridge setup to in situ monitor a four-point bending test on samples of microencapsulated metal dispersed in a dielectric medium. In this setup, the specimen serves as one of the resistors in the Wheatstone bridge circuit. Throughout the four-point bend test, the circuit is monitored with the specimen functioning as one of the bridge arms. The performance of the circuit is evaluated by measuring the normalized bridge voltage.

(5) $$V_{norm}=\frac{\left(V_{h}-V_{\infty }\right)}{\left(V_{o}-V_{\infty }\right)}$$

where V_o_ is the bridge voltage before the damage, V_∞_ is the bridge voltage measured for a fully broken circuit, and V_h_ is the instantaneous bridge voltage of the circuit. The V_norm_ value ranges from zero for a specimen with no electrical conductance to one for a fully conductive specimen. After the fracture process, for each specimen, the efficiency of conductivity restoration, η_c_, is defined as V_norm_.

## 6. Potential Applications of Self-Healing Polymer Composites

All materials, whether organic or manufactured, sustain a decrease during use. If the total amount of damage in the area rises above a certain threshold, a component will fail, and the device’s functionality will be lost. Materials with self-healing capabilities can halt the progression of damage, potentially multiple times. This greatly enhances the lifespan and reliability of the material and, consequently, the device it is used in (damage management). These advanced performance levels are especially important for materials used in fields with limited or no human access, such as medicine and civil, aerospace, automotive, and power engineering sectors. Eliminating monitoring and regulating measures could significantly reduce costs if self-healing of damage induced during manufacturing or application extends the effective lifetime and reliability of new materials. This could lead to substantial resource and energy savings by reducing the previously required safety margins for the design of components exposed to mechanical, thermal, and corrosive stresses in nearly all technical fields. Self-healing materials would greatly enhance material dependability and revolutionize component design and manufacturing. Additionally, extending the lifespan of critical components would significantly improve economic efficiency. For example, extending the lifespan of stents could lower therapy costs. Other potential applications include structures for alternative energy production (wind energy, photovoltaic, solar heat), new lighting technologies (e.g., LEDs), and medical implants. In aerospace and automotive engineering, optimized lightweight design combined with higher structural reliability could reduce energy costs and environmental impact. Arkema’s production of self-healing elastomers and coatings demonstrates the significant application potential of these materials.

Spacecraft commonly utilize composite materials composed of a polymer matrix reinforced with fibers, such as fiber-reinforced plastics (FRPs) with glass fibers (GFRPs), FRPs with carbon fibers, and carbon–epoxy fiber-reinforced plastic (CFRP) composites, including thermosetting epoxy reinforced with high-performance fibers [77]. Other matrix composites used in this context include ceramic matrix composites (CMCs), metal matrix composites (MMCs), and E-glass–epoxy composites (EGCs) [78]. However, these composite materials are prone to deterioration from impact loads. Impact damage begins at the microscopic level with the formation of voids, leading to deep microcracking and delamination within the structure. Traditional damage repair methods, such as resin patches, injection, and thermal plate techniques, have several drawbacks, including inefficiency in addressing invisible damages, the need for continuous monitoring, and limited applicability during construction operations. Additionally, the resulting material often differs from the individual components and lacks a proven track record. As a result, the use of composites in aircraft components has been limited due to their demanding maintenance requirements.

Research on self-healing materials has spanned many academic disciplines and employed various methodologies for almost a decade. While some concepts are driven by direct material applications, understanding the basic mechanisms and principles of self-healing materials remains challenging. Nature has been a significant source of inspiration for developing new self-healing materials and will continue to inspire novel ideas. However, the intrinsic nature of engineered materials must be considered when replicating these models [79,80,81]. Following the repair or restoration of mechanical damage, the mechanical properties are (partially) recovered. Nonetheless, challenges remain, such as creating self-healing materials that can recover from mechanical damage and other adverse effects like intense light and heat, while also regaining conductivity and color. The well-established microcapsule concept could be used to release a charge-transfer salt as a “healing agent” to restore conductivity [82]. Nanostructured systems, such as photonic nanomaterials and metamaterials, also hold promise in this field. In addition to the extensively researched hollow fiber and microencapsulation techniques, thermally triggered healing technologies—such as molecular thermally reversible crosslinks, interdiffusion, and thermoplastic additives—present a new avenue for developing self-healing polymers. These innovations have greater potential to provide multiple healing capabilities. Current advancements are focused on optimizing microvascular healing agent delivery networks and healing agent-filled capsules, which can be used in conjunction with these networks. Reviewing recent developments in self-healing polymeric materials reveals significant progress toward creating genuinely self-healing materials suitable for structural and other commercial applications.

The versatility of polymeric materials allows them to function as self-healing agents in a wide range of applications, from rigid to flexible uses. Various forms of polymeric materials, including pure polymers, polymer composites, and hydrogels, can be utilized for self-healing purposes. The self-healing mechanism has shown considerable improvements in crack properties. However, challenges remain in developing an ideal self-healing system. Future advancements are expected to explore emerging polymeric materials and various self-healing mechanisms for efficiently developing self-healing materials.

Future developments in the field of self-healing will likely focus on biomimetic materials, particularly those with complex molecular aggregates composed of amino acid sequences. Large assemblies of these supramolecular protein-like structures will be able to respond to external stress, mitigating damage, and then self-assemble back to their original form. Working with novel materials made of folded proteins, for instance, should be feasible. These materials could partially unfold to absorb mechanical energy and then refold to self-heal, similar to the behavior observed in self-healing nanobeams.

## 7. Conclusions

This review categorizes repair systems based on recent advancements in self-healing science, with a particular focus on self-healing in composite structures. Self-healing systems can be classified from two main perspectives. The first classification differentiates between intrinsic and non-intrinsic repair systems. The second classification is based on the repair method, dividing self-healing into three major types: microcapsules, vascular networks, and intrinsic systems. The review also attempts to assess repair performance across various mechanical properties, including repair capability and efficiency. Additionally, this review explores the growing trend of research in self-healing materials by examining the number of international publications, active researchers, and the contributions of different scientific disciplines to the development of self-healing technologies. The primary objective is to delve into recent advances with a specific emphasis on the application of self-healing materials in polymer-based composites. In all three mentioned methods, the repair agent is contained within a compartment inside the composite structure. When a crack reaches this area, the compartment ruptures, releasing the repair agent and facilitating repair at the crack tip. Each method has unique characteristics and advantages. Microcapsule-based self-healing is notable for its uniform distribution and ease of design in composite structures compared to other non-intrinsic methods. This review also examines factors influencing the performance of each healing system and the selection of the system type. In the encapsulation method, formaldehyde-based polymers such as urea-formaldehyde are commonly used as the repair agent. While various materials have been employed, research indicates that using the base polymer as a repair agent is often more suitable for repairing the base material than other options.

## Figures and Tables

**Table 1:** Summary of self-healing materials.

Material Type	Characteristics	Application Areas
Microcapsules	Autonomous healing, rupture releases healing agent	Coatings, aerospace, automotive
Thermoplastics	Reversible bonding, phase transformations	Automotive, aerospace, consumer goods
Shape Memory Polymers	Respond to heat, light, mechanical stress	Medical devices, textiles, robotics
Light-Sensitive Materials	Photochemical reactions	Sensors, smart windows, coatings
Hydrogels	Absorb and retain water, flexibility	Medical devices, wound dressings, agriculture

## Data Availability

Data sharing is not applicable to this article as no new data were created or analyzed in this study.
